# 

**Published:** 1964-10

**Authors:** 


					m
V
i fi o ?>
Medic
Jouri
i
Do~l~ (>lf
^^ ? l " V* VTTf : '-'"?*.'? ~T"JV'
Index medicus
1JTC j\ S <Sr 1
| VOL-'.SSU E.
NDEXER .3x3.
THE MEDICAL JOURNAL OF THE SOUTH WEST
w Vol 79 (iv) N? 294

				

